# Major Bleed Post Minimally Invasive Surgical Repair of Inguinal Hernia

**DOI:** 10.7759/cureus.9940

**Published:** 2020-08-22

**Authors:** Anupam K Gupta, Monica I Burgos, Antonio J Santiago Rodriguez, Miguel Lopez-Viego, Matthew M Ramseyer

**Affiliations:** 1 Minimally Invasive Surgery, University of Miami Hospital, Miami, USA; 2 Internal Medicine, Universidad Autonoma de Guadalajara, Guadalajara, MEX; 3 Surgery, University of Medicine and Health Sciences, Puerto Rico, USA; 4 Surgery, Bethesda Hospital East, Boca Raton, USA; 5 Surgery, Bethesda Hospital, Boynton Beach, USA; 6 Trauma and Critical Care Surgery, Florida State University Collage of Medicine, Palm Beach, USA

**Keywords:** corona mortis, inguinal hernia, minimally invasive laparoscopy, bleeding, atypical vessel, hemoperitoneum

## Abstract

At our center, over 200 patients undergo minimally invasive repairs of inguinal hernias every year using a laparoscope and a robotic-assisted technique. In three patients who underwent a minimally invasive procedure for uncomplicated indirect inguinal hernia, the postoperative course was complicated with bleeding and required an exploratory laparotomy. Post-procedure, the patients developed tachycardia and hypotension with a drop in hemoglobin, for which the patients required surgical re-exploration in an emergent fashion. The factors leading to bleeding were analyzed and are reported herein to increase awareness and prevent the occurrence of these complications during inguinal hernia surgery.

## Introduction

Inguinal hernias are one of the most common hernias seen in daily practice [1]. This pathological condition manifests as a bulge in the groin. Surgery for an inguinal hernia can be an open or minimally invasive technique performed with a laparoscope or robot [1]. Although there is increasing use of minimally invasive procedures to repair inguinal hernias, they can lead to severe complications if the surgeon is not careful. Two different minimally invasive techniques with a laparoscopic or robotic approach include transabdominal preperitoneal (TAPP) repair and total extraperitoneal (TEP) repair [1]. This case report evaluates the root cause analysis of three patients who developed massive bleeding after minimally invasive TAPP inguinal hernia repair.

## Case presentation

Three patients (Table 1) who underwent minimally invasive surgery for repair of indirect inguinal hernia experienced postoperative bleeding. Each patient presented with an asymptomatic uncomplicated indirect inguinal hernia and requested a minimally invasive approach for its repair. All three patients underwent mesh repair of their indirect inguinal hernia. Blood thinners had been previously prescribed for two of the patients, who abstained from taking them for five days before surgery, and aspirin therapy patients continued aspirin use until the day of their scheduled surgery. All patients had Foley catheters intraoperatively placed, which were removed at the completion of the surgery. The patients were placed supine, with both arms tucked in, and in the Trendelenburg position. A Foley catheter was placed after the induction of anesthesia and was removed at the end of the surgery. Dissection was performed using scissors, Bovie, and blunt graspers, depending on the surgeon's choice. The choice of mesh was also surgeon-dependent. The surgical technique for robotic-assisted (daVinci®; Intuitive Surgical, Sunnyvale, CA) and laparoscopic surgery used three ports. The first port was 10 mm long and was positioned at a supraumbilical site in the midline, and two additional ports, each 5 mm long, were on either flank. The laparoscopic technique also required the placement of three ports at similar locations.

**Table 1:** Patient presentations Abbreviations: HTN= Hypertension, HLD= Hyperlipidemia, DM= Diabetes mellitus, ASA= Aspirin, PMH= Past Medical History, M= Male, TAPP= Transabdominal Preperitoneal Repair

Age (years)/sex	PMH	Blood thinners	Hernia	Technique (TAPP)	Etiology
77/M	HTN, DM, HLD	ASA, coumadin	Right indirect inguinal	Robotic	Corona mortis
50/M	HTN, DM	ASA, coumadin	Right indirect inguinal	Laparoscopic	Not seen
45/M	None	None	Left indirect inguinal	Laparoscopic	Inferior epigastric

These three patients underwent TAPP repair of their indirect inguinal hernia. In surgery, dissection was initiated by retracting the peritoneum from the lateral to medial aspect to repair the indirect inguinal hernia. The plane of the preperitoneal space of Retzius and Bogros was accessed. Subsequently, anatomical landmarks were identified, including the pubis and Cooper's ligament medially and the psoas muscle laterally. In the laparoscopic technique, the mesh was secured in place with the help of absorbable tackers.

In the immediate postoperative recovery, there was evidence of tachycardia and hypotension for all three patients. After the patients were clinically evaluated, they were subsequently returned to the operating room. A midline incision was utilized to enter the abdominal cavity, and upon exploration, hematomas and clots were removed before looking for a source of bleeding. In one patient, it was evident that the bleeding source was from the left inferior epigastrium, and ligation of the inferior epigastric artery was subsequently performed. The other two patients developed bleeding in the preperitoneal space, which required emergent evacuation. The corona mortis was the source of the bleeding in one of these two patients, and it was ligated; bleeding was controlled with hemostatic agents. In the other patient, there was no evidence of an active bleeder, and therefore, multiple hemostatic agents were placed in the preperitoneal space. After controlling hemostasis, the mesh repair remained intact.

## Discussion

Inguinal hernia repairs are among the most common procedures performed by surgeons in the United States [1]. Groin hernias can be indirect, direct, or femoral-based in location [1]. An open, robotic, or laparoscopic approach can help to provide a tension-free repair [1]. Because a generally accepted technique suitable for all inguinal hernia repairs does not exist, surgeons should offer both an anterior open and a posterior laparo-endoscopic (TEP or TAPP) approach. Laparoscopic TAPP repair treatment of hernias has demonstrated several benefits when compared to the conventional surgery approach. These benefits include more optimal cosmesis, less postoperative pain, and faster resumption of physical, professional, and sports activities [2,3]. The preperitoneal space is largely avascular and is composed of adipose tissue, loose connective tissue, and membranous tissue. 

Even if laparoscopic hernia repair can be performed in a minimally invasive manner, it can present with intraoperative and postoperative complications. Postoperative complications include hematomas, pain in the thigh and scrotum, and emphysema [4]. Other more common complications are mentioned in Table 2.

**Table 2:** Most common complications of laparoscopic hernia repair

Vessel injury	Bowel injury/urinary retention
Inguinal hematoma/seroma	Ischemic orchitis
Testicular edema	Hydrocele
Infections	Neuralgia due to nerve damage

The preperitoneal retropubic space is in the midline of the lower abdomen with the superficial transverse fascia and the pubic bone anteriorly, the bladder posteriorly, the umbilicus level superiorly, the pelvic floor muscles inferiorly, and the inferior epigastric arteries laterally [5]. Bleeding during inguinal hernia can occur due to vessel dissection, improper port placement, or trauma during fixation of the mesh with tackers [4]. Bleeding can occur intraoperatively from the cremasteric artery, the internal spermatic artery, branches of the inferior epigastric vessels, deep circumflex artery and the external iliac vessels, iliac circumflex vessels, and obturator vessels [6,7]. Careful surgical technique with attention to proper hemostasis and pressure generally obviates the need for formal vascular intervention. 

Some anatomical structures are challenging to visualize and may be injured if the surgeon is not careful. One such structure includes the artery of Sampson. Currently, three confirmed cases of hemoperitoneum due to accidental laceration of this artery have been previously published. Sampson's artery, which occurs in female patients, originates from the arcade formed between the uterine and ovarian artery [8]. As Hebert et al. note, the “disruption of Sampson's artery can lead to hemoperitoneum following ligation of the uterine round ligament” [8]. Another vessel that is not commonly injured and could be challenging to visualize is the corona mortis. Injury to the corona mortis can result in massive hemorrhage resulting from intraoperative injuries [9]. The corona mortis is a communicating vessel between the obturator and external iliac vessels and traverses posterior to the superior pubic ramus at a variable distance from the symphysis pubis [10]. The corona mortis includes arteries and veins, most of which travel alone and leave the pelvic cavity via the obturator canal [5]. The artery and vein visibility in the corona mortis can increase from 1.0% and 28.4% to 31.0% and 46.7%, respectively, by decreasing the pneumoperitoneum pressure from 14 mmHg to 8 mmHg [7]. 

Injury to the inferior epigastric vessels is relatively uncommon. Still, it may occur in up to 2% of cases during insertion of trocars or widening of an incision that is performed to allow passage of a cannula or removal of tissue from the peritoneal cavity [11]. To reduce the risk of injury, we suggest the careful placement of ports. Early recognition of a vascular injury is essential because a delay in the diagnosis is a significant contributor to postoperative morbidity and mortality. Arterial injury is more easily recognized during the TEP procedure; however, venous injury can be masked by the sealing effect of the pressure of insufflated carbon dioxide, which presented with postoperative venous hemorrhage in one of our described patients [12].

Adequate pneumoperitoneum is essential for visualization of the intraabdominal organs and the performance of laparoscopic procedures in which the standard insufflation pressures range from 8 mmHg to 10 mm Hg [13]. One can increase the corona mortis visibility by decreasing the intraabdominal pressure from 14 mmHg to 8 mmHg [12]. At a pressure of 14 mmHg, venous structures are not prominent and thus present a risk for predisposing the vessels to injury [12]. The pre-peritoneal space is deflated under direct vision, which ensures that the reduced peritoneum and fatty tissues remain above the prosthetic material [14]. After the laparoscopic procedure, all cannula sites should be visualized from within the peritoneal cavity to exclude the presence of active bleeding and routinely reduce the pneumoperitoneum at the end of dissection for venous bleeding [14]. Pressures of insufflated carbon dioxide more than 10 mmHg in the preperitoneal space during TEP hernia repair hinder the correct identification of vessels on the retropubic surface [12]. Some studies propose decreasing the pneumoperitoneum pressure to 8 mmHg only before the fixation of mesh and providing a more reliable identification of the vessels, especially veins and fragile arteries, to prevent the potential risk of injury. 

## Conclusions

Although it is common to use minimally invasive techniques to repair an inguinal hernia, they can result in certain complications. Post-surgical bleeding can be severe, requiring additional surgery and prolonged hospital stay. This can be prevented by meticulously performing inguinal hernia repair techniques. To avoid the occurrence of post-surgical bleeding, we now routinely reduce pneumoperitoneum at the end of dissection to examine venous bleeding. At this point, we also instruct the patients to lay flat so that we can observe them for a few minutes and detect any bleeding. Dissection below Cooper's ligament is minimal, and, if needed, blunt dissection is not performed to avoid tearing thin-walled veins. Lastly, to prevent complications, the placement and removal of lateral ports should occur under visualization, and the inferior epigastric artery should also be visualized if possible.
